# MRI-derived brain iron, grey matter volume, and risk of dementia and Parkinson’s disease: Observational and genetic analysis in the UK Biobank cohort

**DOI:** 10.1016/j.nbd.2024.106539

**Published:** 2024-05-22

**Authors:** Francesco Casanova, Qu Tian, Daniel S. Williamson, Yong Qian, David Zweibaum, Jun Ding, Janice L. Atkins, David Melzer, Luigi Ferrucci, Luke C. Pilling

**Affiliations:** a Department of Clinical and Biomedical Sciences, University of Exeter, Magdalen Road, Exeter, Devon EX1 2LU, UK; b Translational Gerontology Branch Longitudinal Studies Section, National Institute on Aging, 251 Bayview Blvd., Suite 100, Baltimore, MD 21224, USA; c Department of Medical Imaging, University of Exeter, Magdalen Road, Exeter, Devon EX1 2LU, UK

**Keywords:** Iron, Dementia, Parkinson’s, Genetics, Grey matter

## Abstract

**Background:**

Iron overload is observed in neurodegenerative diseases, especially Alzheimer’s disease (AD) and Parkinson’s disease (PD). Homozygotes for the iron-overload (haemochromatosis) causing *HFE* p.C282Y variant have increased risk of dementia and PD. Whether brain iron deposition is causal or secondary to the neurodegenerative processes in the general population is unclear.

**Methods:**

We analysed 39,533 UK Biobank participants of European genetic ancestry with brain MRI data. We studied brain iron estimated by R2* and quantitative susceptibility mapping (QSM) in 8 subcortical regions: accumbens, amygdala, caudate, hippocampus, pallidum, putamen, substantia nigra, and thalamus. We performed genome-wide associations studies (GWAS) and used Mendelian Randomization (MR) methods to estimate the causal effect of brain iron on grey matter volume, and risk of AD, non-AD and PD. We also used MR to test whether genetic liability to AD or PD causally increased brain iron (R2* and QSM).

**Findings:**

In GWAS of R2* and QSM we replicated 83% of previously reported genetic loci and identified 174 further loci across all eight brain regions. Higher genetically predicted brain iron, using both R2* and QSM, was associated with lower grey matter volumes in the caudate, putamen and thalamus (e.g., Beta-putamenQSM: −0.37, *p* = 2*10–46). Higher genetically predicted thalamus R2* was associated with increased risk of non-AD dementia (OR 1.36(1.16;1.60), p = 2*10–4) but not AD (*p* > 0.05). In males, genetically predicted putamen R2* increased non-AD dementia risk, but not in females. Higher genetically predicted iron in the caudate, putamen, and substantia nigra was associated with an increased risk of PD (Odds Ratio QSM ~ substantia-nigra 1.21(1.07;1.37), *p* = 0.003). Genetic liability to AD or PD was not associated with R2* or QSM in the dementia or PD-associated regions.

**Interpretation:**

Our genetic analysis supports a causal effect of higher iron deposition in specific subcortical brain regions for Parkinson’s disease, grey matter volume, and non-Alzheimer’s dementia.

## 1. Introduction

Brain iron deposition can adversely affect brain function through neuroinflammation (Ward et al., 2022), and has been observed in neurodegenerative diseases including Alzheimer’s disease (AD) and Parkinson’s disease (PD) (Han et al., 2021; Ayton and Lei, 2014). We previously found that genetically predicted higher serum iron increased risk of dementia (Casanova et al., 2024), and dementia is more common in people homozygous for *HFE* p.C282Y, which causes the iron-overload disease hemochromatosis (Atkins et al., 2021). Yet the limited efficacy of de-ironing drugs in clinical trials involving patients with dementia and PD (Galasko and Simuni, 2022) supports a secondary role of iron. Therefore, whether iron deposition is the cause or consequence of neurodegenerative process remains unclear.

Brain iron can be estimated using magnetic resonance imaging (MRI); R2* and quantitative susceptibility mapping (QSM) provide numerical values of tissue susceptibility (the magnetisation that occurs when a substance experiences a magnetic field). Genome-Wide Association Studies (GWAS) have discovered genetic variants associated with MRI estimated iron in the brain (Wang et al., 2022), as well as variants associated with risk of dementia and PD. (Kunkle et al., 2019; Nalls et al., 2019) We can gain insight into the direction of effect between brain iron and neurodegeneration using genetics by testing the hypothesis that individuals carrying a greater number of brain iron-increasing genetic variants (i.e., lifelong exposure to higher iron) have higher risk of dementia or PD. This method, known as Mendelian Randomization (MR), is a statistical approach to use the MRI R2* and QSM-associated genetic variants as instrumental variables to estimate the causal effect of brain iron on disease risk, free from many traditional confounding factors, though with specific assumptions: 1) the genetic variants are associated with the exposure, 2) the genetic variants should not associate with confounders and 3) the genetic variants do not affect the outcome other than through the exposure. (Sanderson et al., 2022) In the opposite direction, MR can be used to test whether dementia or PD-associated genetic variants increase MRI-estimated brain iron, which would support iron deposits are secondary to the disease process.

We aimed to estimate the association between genetically predicted brain iron-related phenotypes with dementia – including AD and not-AD dementia – and PD. We also estimated associations with MRI grey matter volumes. We also investigated whether iron accumulation is secondary to the disease process by testing if higher genetic liability to AD or PD is associated with higher brain iron. UK Biobank MRI brain iron data is now available from both R2* and QSM methods (Wang et al., 2022) in up to 42,000 participants, providing new opportunities for exploring our aims.

## 2. Methods

### 2.1. Study sample

UK Biobank (UKB) is a cohort study of ~502,000 UK adults aged 40–70 years at baseline assessment (2006 to 2010). Data includes baseline characteristics, biomarkers, genetics, and linked medical records, with a subgroup of participants having undertaken an additional MRI visit (current data 2014 to 2022).

UKB data are available to any bone fide researcher following application. The Northwest Multi-Center Research Ethics Committee approved the collection and use of UKB data (Research Ethics Committee reference (Bycroft et al., 2018)/NW/0382). Participants gave informed consent for the use of their data, health records, and biological materials for health-related research purposes. Access to UKB was granted under Application Number 83534.

### 2.2. MRI imaging measures

The UKB MRI data has been processed centrally and imaging-derived phenotypes (IDPs) made available to analysts. In this study we used R2* relaxation and QSM methods (Wang et al., 2022), that estimate brain iron deposition, available in 8 subcortical regions: the nucleus accumbens, amygdala, caudate, hippocampus, pallidum, putamen, substantia nigra, and thalamus. We also analysed regional grey matter volumes (from the T1 FAST protocol) in the same areas, except where the regions were not available (accumbens and substantia nigra; see Supplementary Information for exact data fields). Mean values for right and left hemisphere measures were used (we therefore analysed 8 R2* IDPs, 8 QSM IDPs, and 6 grey matter IDPs).

### 2.3. Disease outcomes: dementia and Parkinson’s

We used a combination of summary statistics from published GWAS studies, where available, and GWAS in UKB when a published GWAS was not available. For AD, we used the GWAS published by Kunkle et al. (Kunkle et al., 2019) For PD, we used the published GWAS by Nalls et al. (Nalls et al., 2019) For all-cause dementia (ACD) and non-AD dementia no large published GWAS were available at the time of analysis. We therefore performed GWAS in UKB. Diagnoses of ACD and AD were ascertained from the hospital inpatient record, primary care data (where available), and death record (see Supplementary Methods for details on diagnostic codes). We derived the non-AD dementia outcome by excluding participants who received an AD diagnostic code from the ACD cases (the reference group were participants with no dementia diagnosis).

### 2.4. Genotype data and genome-wide association studies

We used imputed genotype information (Bycroft et al., 2018) on 16 million genetic variants in 487,442 participants, after excluding variants with <0.1% minor allele frequency or with imputed INFO score < 0.3 (see Supplementary Information for details). GWAS were performed in up to 451,000 participants genetically-similar to the 1000 Genomes EUR population (described previously) (Casanova et al., 2024). For GWAS of 17 IDPs (8 brain R2* IDPs and 8 brain QSM IDPs) we used BOLT-LMM (v2.3.2) (Loh et al., 2015) to estimate associations, adjusted for age at imaging assessment, sex, imaging assessment centre, and genotyping microarray, with genetic relatedness and population structure adjusted for by the BOLT-LMM random-effects model. IDPs were inverse-rank transformed prior to analysis. For GWAS of binary outcomes (AD and non-AD dementia) we used SAIGE-LMM (v0.35.8) (Zhou et al., 2018), adjusted for age at baseline assessment, sex, assessment centre, genotyping microarray, and genetic related and population structure. Analyses were repeated, stratified by sex. We looked for previously reported loci using Phenoscanner in R (https://github.com/phenoscanner/phenoscanner).

For Mendelian randomization (MR) analysis (described below) we use genetic variants as instruments (proxies) for the exposures and outcomes to estimate the causal effect. We identified the genetic instrumental variables based on *p*-values <5*10 ^−8^ and distance (>500 kb from nearest IV, Supplementary table 2).

To test for bias due to population stratification and estimate SNP-based heritability we used LD Score Regression (LDSC) v1.0.1 (Bulik-Sullivan et al., 2015).

### 2.5. Statistical analysis - observational

We investigated factors associated with brain R2* and QSM using multivariable linear regression for all available brain areas. Common dementia, PD and cardiovascular risk factors were used in the model, in detail we tested the association with: age at MRI assessment, sex, UK Biobank assessment centre, APOE e4 status (defined by rs429358 and rs7412), body mass index (BMI), smoking (current, former, never), educational attainment (none, primary, secondary, college, University or equivalent), hypertension and diabetes.

### 2.6. Statistical analysis - Mendelian randomization

To estimate the causal effects between exposures and outcomes we used previously published MR methods, with the primary estimate from Inverse-Variance Weighted (IVW) analysis. We used the TwoSampleMR R package (v0.5.7 - github.com/MRCIEU/TwoSampleMR). IVW MR analysis is susceptible to bias known as “Winner’s curse” if the exposure and outcome instruments are selected using overlapping samples. Where this was the case in our analysis (e.g., between UKB brain iron and UKB dementia) we used the MRlap R package (v0.0.3.1 - github.com/n-mounier/MRlap) to estimate the IVW causal effect, adjusted for sample overlap and weak instrument bias (Mounier and Kutalik, 2023). See Supplementary Information for more details on the assumptions and sensitivity analyses performed.

Mendelian randomization relies on specific assumptions: 1) the genetic variants are associated with the exposure, 2) the genetic variants should not associate with confounders and 3) the genetic variants do not affect the outcome other than through the exposure (Sanderson et al., 2022).

The IVW method estimates the causal effect of the genetically predicted exposures on the genetically predicated outcomes 2. The IVW assumes there is balanced horizontal pleiotropy (i.e. with a zero mean); we therefore additionally applied ‘weighted median’ (assumes <50% of the weight in the analysis comes from invalid instruments) and ‘MR-Egger’ (allows for unbalanced pleiotropy providing genetic variants’ effect on the exposure is not correlated with their pleiotropic effects on the outcome) approaches to test robustness of the estimate. The MR-Egger method additionally estimates an intercept and deviation from the null is used to test for possible unbalanced pleiotropy.

## 3. Results

In the analysis of UK Biobank (UKB) participants of EUR-like genetic ancestry (*n* = 451,231), 8040 participants (1.78%) were diagnosed with all-cause dementia (ACD) during the follow up (hospital inpatient records, primary care data, and death record, up to 16.6 years after baseline assessment). Summary statistics for the participants are presented in Table 1.

### 3.1. Observational associations between brain iron estimates in 8 regions

**Fig. 1.**
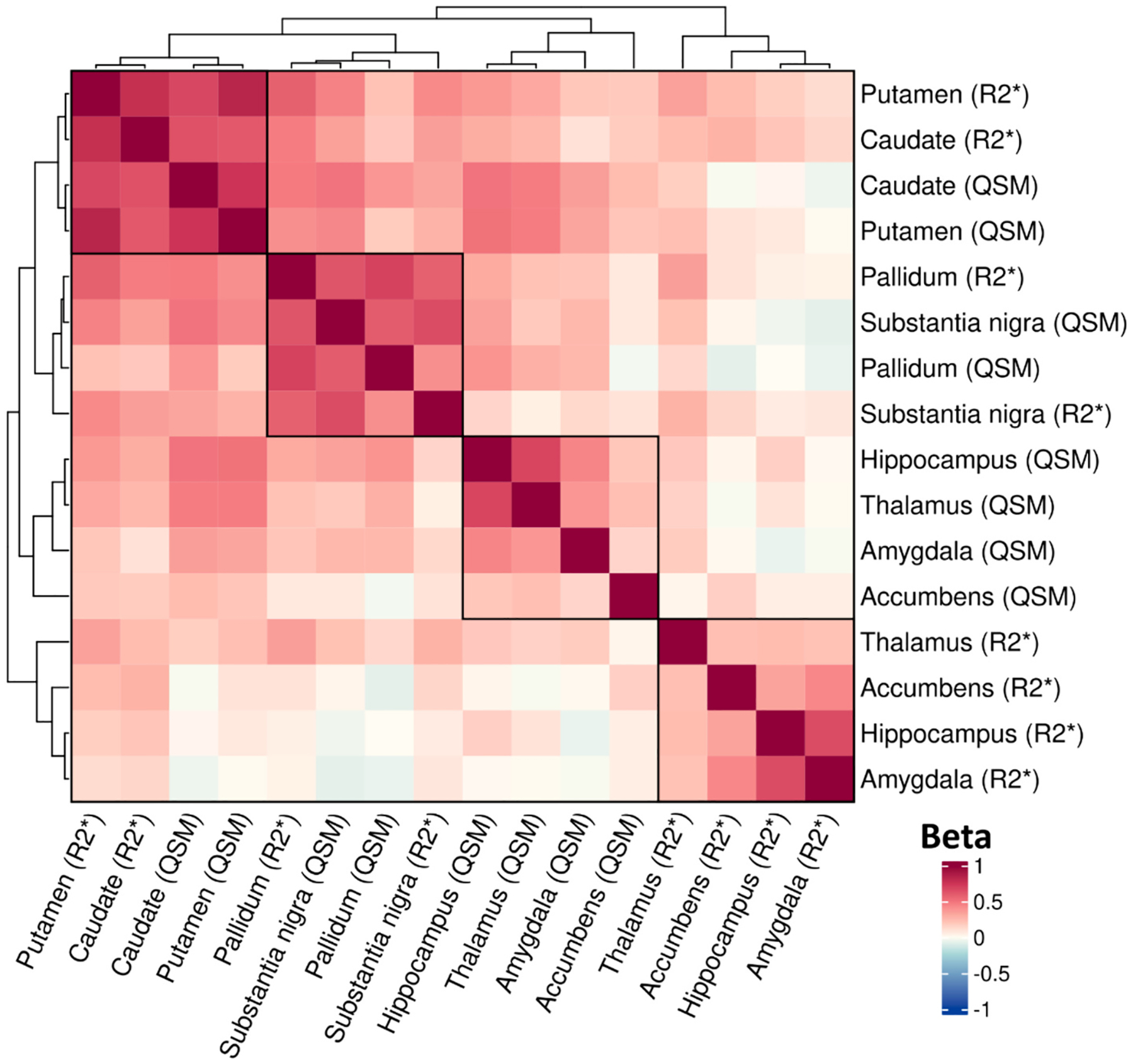
Observational associations between brain iron estimates of R2* and QSM in 8 brain regions. Heatmap showing associations between brain R2* with brain QSM. Estimates from linear regression models in 39,533 UK Biobank participants, adjusted for age at MRI assessment, sex, and MRI assessment centre. Beta = standardized coefficient (per standard deviation). Ordered by hierarchical clustering, from which four clear groups are highlighted by overlaid squares. Details in Supplementary Table 3.

A subset of 39,533 participants had brain MR Imaging Derived Phenotypes (IDPs) at the time of analysis, with R2* and QSM estimated in 8 regions (higher values indicate greater iron deposition). Overall, the intra-region R2* and QSM estimates tended to be higher than inter-region correlations (Fig. 1), with the putamen and caudate exhibiting strong associations (beta-_QSM_ 0.83, 95% Confidence Intervals [CI] 0.821 to 0.833, *p* = 1*10^−15,390^), whereas for regions such as the hippocampus the associations between the R2* and QSM estimates was lower (beta 0.17: 95% CIs 0.16 to 0.18, p = 1*10^−257^). See Supplementary Table 3 for details.

### 3.2. Genetic analysis of brain iron estimates in 8 regions

GWAS of 16 IDPs (both R2* and QSM in 8 regions) identified 250 loci in total. Our GWAS results were similar to the previously published GWAS of the same phenotypes (Wang et al., 2022) with 83% of the signals reported confirmed here. Across all the 16 R2* and QSM IDPs we analysed, we identify a total of 174 loci not previously reported to be associated with any R2* or QSM IDP in Wang et al. (Wang et al., 2022) Full summary statistics are available to download from FigShare (https://doi.org/10.6084/m9.figshare.25343170). The lead variant for each locus, for each IDP GWAS, is listed in Supplementary Table 2.

Our results confirm the previously reported pathways such as iron transport and homeostasis (e.g., *HFE, SLC40A1, TF, TFRC*), metal ion transporters (*SLC39A8, SLC39A12*), calcium homeostasis (*CACNB2, TPCN1, BANK1*) and glia and myelin (*MOBP, GFAP*). Results from Phenoscanner (Supplementary Tables 4 and 5) revealed that previously reported individual SNPs were most frequently associated with height, haematology parameters (i.e., Mean corpuscular volume, Mean corpuscular haemoglobin etc), BMI, blood pressure, body composition parameters (impedance, fat mass, fat-free mass) and bone density.

We used the independent genetic variants identified for each IDP as instrumental variables (IVs) in our Mendelian randomization (MR) analyses described below (where we refer to the “genetically predicted IDP”). For the exposures associated with dementia or Parkinson’s outcomes we formally tested the validity of the IVs used (also see below).

### 3.3. Associations between estimated brain iron and grey matter brain volumes: observational and genetic results

**Fig. 2.**
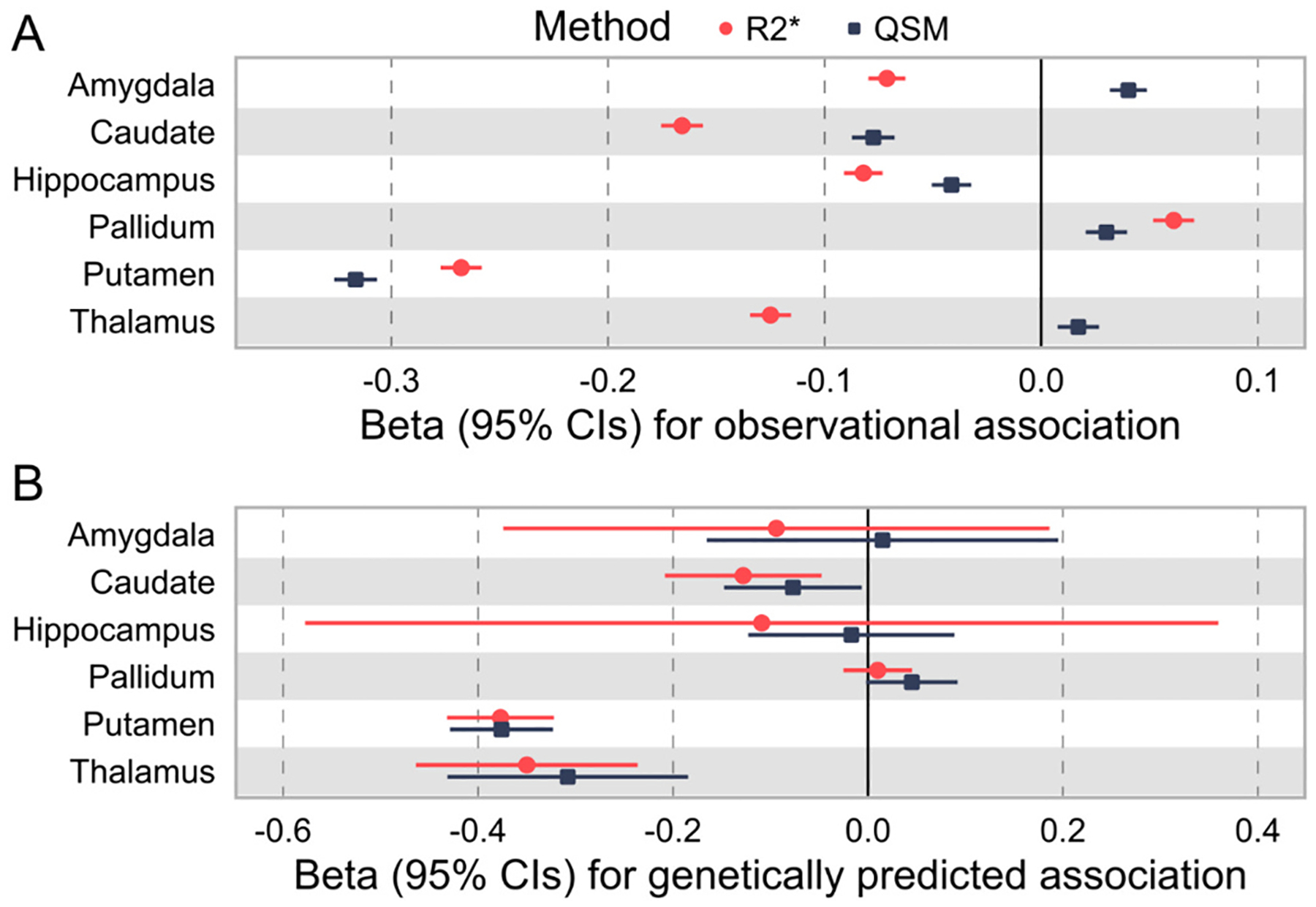
Associations between brain iron estimates and brain volume, observationally (panel A) and genetically predicted (panel B). A) Forest plot showing associations between brain R2* and brain QSM (higher values indicates more iron), with grey matter volume in the same region. Estimates from linear regression models adjusted for age at MRI assessment, sex, MRI assessment centre, and total grey matter volume. B) Forest plot showing Mendelian randomization analysis using genetic variants associated with brain iron to estimate the causal effect of brain iron in different regions on grey matter volume. See Supplementary Tables 6 and 7 for details. Beta = standardized coefficient (per standard deviation effect). CIs = confidence intervals. ** = significant association after adjustment for 6 tests (*p* < 0.0083) (panel B only – all associations in panel A are p < 0.0083).

Observationally, IDPs for R2* and QSM in 8 regions were strongly associated with IDPs for grey matter volumes in the same regions (Fig. 2A). The largest effect was for putamen QSM, with higher estimated brain iron associated with smaller grey matter volume in the putamen (beta −0.31, 95% CI −0.32 to −0.30, *p* = 9*10^−845^), in linear regression models adjusted for age, sex, and assessment centre. The direction was not always consistent across regions, for instance higher QSM and R2* in the pallidum was associated with larger grey matter volume in the pallidum (beta_-R2*_ 0.06, 95% CIs 0.05 to 0.07, *p* = 8*10^−34^). See Supplementary Table 6 for details.

Genetically predicted IDPs for R2* and QSM were associated with lower grey matter volumes in putamen and thalamus (Fig. 2B and Supplementary table 7). In the caudate R2* was associated with lower grey matter volume but QSM *p* value was higher than the Bonferroni multiple test correction threshold (8 tests, *p* = 0.006). All other areas showed no association after multiple test correction.

### 3.4. Genetically predicted estimated brain iron depositions and risks of PD and dementia

**Fig. 3.**
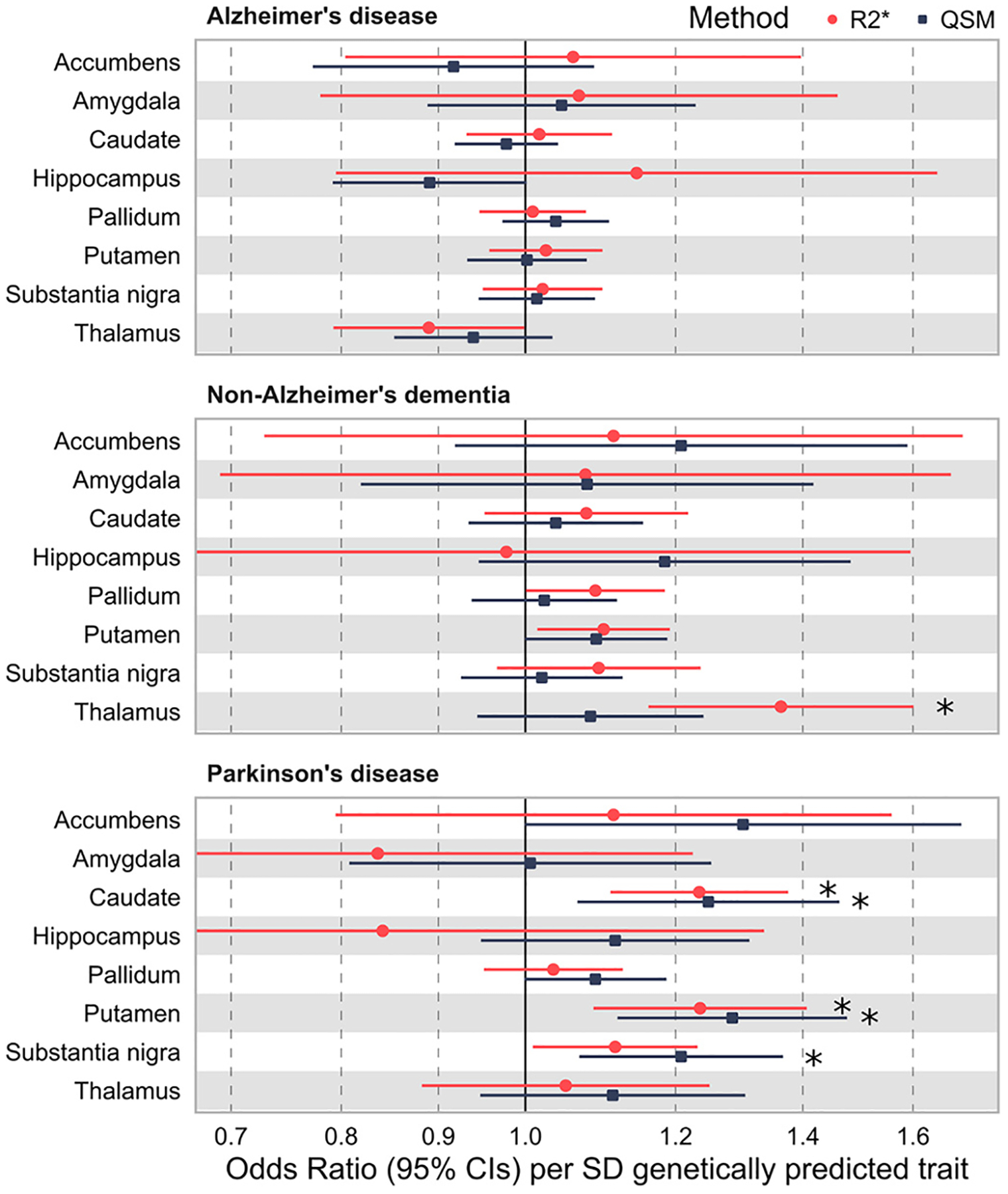
Associations of genetically predicted brain iron with Alzheimer’s disease [AD], non-AD dementia, and Parkinson’s disease (Mendelian randomization). Mendelian randomization analysis using genetic variants associated with brain R2* and QSM to estimate the causal effect of brain R2* and QSM in different regions on risk of dementia (AD and non-AD) and PD. X-axis limits set from 0.7 to 1.7 for clarity. Details in Supplementary Tables 8 and 11. * = significant association after adjustment for 8 tests (*p* < 0.0063). Genetic variant associations with AD are from Kunkle et al. 8; genetic variant associations with PD are from Nalls et al. 9; genetic variant associations with non-AD are from our GWAS in UK Biobank.

Genetically predicted R2* and QSM in caudate, putamen and substantia nigra were associated with higher odds of PD (Fig. 3 and Supplementary table 8). The associations for caudate R2*, substantia nigra QSM and accumbens R2* did not remain significant after multiple test correction and we found no associations for other regions.

### 3.5. Continued

Genetically predicted iron deposition in the thalamus was associated with increased odds of not-AD dementia (R2* OR 1.36, 95%CI 1.16 to 1.60) using the R2* method with QSM results directionally consistent but not significant (Fig. 3 and Supplementary tables 9 and 10). Other associations did not remain significant after multiple test correction, and we found no associations with AD for any region.

Genetically predicted R2* and QSM in putamen was nominally associated with increased odds of non-AD dementia (R2* Odds Ratio [OR] 1.128, 95% CI 1.058 to 1.238; QSM OR 1.147, 95%CI 1.055 to 1.247, Fig. 3 and Supplementary table 9 and 10) but not after multiple testing correction (8 brain phenotypes, *p* = 0.006).

We tested MR assumptions and found that IVs used here were strongly associated with R2* or QSM (median F-statistic 41.4, no variant with F < 10, MR assumption 1, Supplementary table 2). We also tested whether genetically predicted brain iron was associated with non-AD dementia through known vascular pathways (BMI and coronary artery disease; MR assumption 2) and found no association (Supplementary table 11). This supports the validity of the brain iron IVs (they not associated with the vascular pathways tested). We further investigated the predictors of R2* or QSM using the observational data. In linear regression models we found that age, sex, BMI, smoking status and type-2 diabetes were associated with R2* and QSM in all regions analysed (Supplementary Table 14). After adjustment for multiple testing, the BMI-substantia nigra R2* and diabetes–thalamus R2* associations became non-significant. Hypertension was associated only with Thalamus QSM. *APOE* e4 homozygosity was not associated with R2* or QSM.

There was evidence of pleiotropy from MR-Egger (Supplementary table 9 and 10) further discussed in the sensitivity section below.

### 3.6. Genetic liability for Alzheimer’s disease and Parkinson’s disease and associations with brain iron estimates

After adjustment for multiple testing (Bonferroni *p*-value threshold for 8 tests = 0.006), genetical liability to AD was significantly associated with higher QSM in the amygdala (beta-IVW 0.026, 95% CI 0.012 to 0.040, *p* = 0.0005). Genetic liability to AD was nominally associated with higher R2* in the thalamus (beta-IVW 0.019, 95% CI 0.001 to 0.037, *p* = 0.03). See Supplementary Table 12 for details. Genetical liability to PD was not significantly associated with R2* or QSM after adjustment for multiple testing, though there was a nominal association in the accumbens, where higher genetic liability to PD was associated with lower QSM (beta-IVW −0.033, 95% CI −0.058 to −0.008, *p* = 0.01). See Supplementary Table 13 for details.

### 3.7. Sex stratified analysis

Sex stratified analysis revealed that the association between genetically predicted brain iron and grey matter volume was different between males and females in hippocampus (Fisher’s Z *p* = 0.032) using QSM but not R2*. No sex differences were observed for other areas (Supplementary table 7). R2* and QSM in putamen and the risk of not-AD dementia was stronger in males than females using both QSM and R2*. We also found significant sex differences, with significant associations in males and not in females, in hippocampus using QSM and pallidum using R2* with all-causes dementia.

### 3.8. Sensitivity analysis

We used MRlap to evaluate whether the genetically predicted brain iron estimates of R2* and QSM associations with grey matter volumes as well as dementia were biased by sample overlap or weak instrument bias and found no evidence to support any bias (Supplementary Table 15). We conducted “leave one out” analysis for all traits showing an association with grey matter and neurodegenerative diseases after multiple test correction (Supplementary table 16). For outcome grey matter association removing SNPs had no effect except for rs10430577 and rs668799 for caudate QSM. For outcome PD leave one out analysis did not change the results of the PD’s analysis for any of the main analysis significant exposures except rs73090631 for Accumbens QSM. For outcome dementia all-causes and not-AD dementia removing SNPS had no effect for thalamus R2 except rs1800562 and all-causes dementia (Supplementary table 16).

There was evidence of pleiotropy for Thalamus R2* to dementia all-causes and not-AD dementia as well as putamen QSM and R2* on grey matter volume (MR assumption 3). I-squared was between 0.980 and 0.987 for all outcomes (Supplementary Table 10 and 17). Inspection of the individual effects plots (see Supplementary Figs. 1 to 4) revealed that the variant driving the significant Egger intercept in MR (rs7113219) is biasing our results towards the null (see also leave one out Supplementary table 16). There was evidence of heterogeneity for all our exposures indicating multiple biological mechanisms (Supplementary Table 18); we performed sensitivity analyses testing for pleiotropy, and also additional analyses to increase confidence that the main conclusion is not confounded by vascular risk factors (described in section 3.4).

## 4. Discussion

We found that genetically predicted MRI-derived brain iron deposition (R2* and QSM) in specific regions was associated with grey matter atrophy and increased risk of PD and non-AD dementia, but not AD. We found little evidence for a bi-directional effect; genetic liability to AD or PD did not increase brain iron in the same regions. Previous research found increased iron deposits in brain tissue of patients with neurodegenerative diseases like dementia and PD, (Ward et al., 2022; Ward et al., 2014) but whether this is causal or secondary to the disease processes was unknown. Our results are consistent with a causal effect of brain iron deposition (R2* and QSM in specific regions) on risk of PD and non-AD dementia, especially in males, rather than secondary to the diseases process. We also show that genetically predicated brain iron reduced grey matter volume in the basal ganglia, consistent with longitudinal studies of this brain region (Daugherty and Raz, 2016). Grey matter atrophy is a hallmark of cognitive impairment, and together our data supports a role of brain iron in this pathway leading to dementia.

We found a casual effect of genetically predicted R2* in the thalamus on non-AD dementia. Other areas were not statistically significant overall, however in sex-stratified analyses we found a significant association between QSM and R2* in putamen and non-AD dementia in males but not in females, in addition to male-only associations in hippocampus and pallidum with all-cause dementia. A sex difference is not unexpected in iron related phenotypes and is consistent with findings that iron overload impacts males more than females (Adams et al., 2023). It is possible that some of the areas might become statistically significant with increased power, such as with further releases of UKB MRI data in future years. Additionally, null associations – such as our observation that neither genetically predicted R2* or QSM in the substantia nigra increased dementia risk – does not rule them out: measurement error or low absolute iron deposition reduces power to detect associations, and iron in these regions may therefore still be important for disease pathology.

We found no association between any brain iron estimate and AD dementia, consistent with our previous report of no association between genetically predicted serum iron and AD dementia (Casanova et al., 2024), but in contrast with studies suggesting a role of brain iron in the development of amyloid plaques (Peters et al., 2015). Brain regions available in UKB were limited to subcortical areas, and while the lack of association between iron deposition in the hippocampus and AD is unexpected, it is possible that iron in cortical areas might show an association, as cortical iron is thought to be key to cognitive deterioration in AD (Ayton et al., 2020).

We found an association between genetically predicted R2* and QSM in caudate, substantia nigra and putamen and PD. All these areas have been suggested to be involved in the pathological development of PD. Our data support the large body of evidence showing iron accumulation in the substantia nigra in postmortem studies of PD patients (Snyder and Connor, 2009), as well as some evidence of increased iron deposition in the putamen (Wallis et al., 2008) and caudate (Rossi et al., 2014) even if some studies found no difference in iron accumulation in putamen and caudate (Dexter et al., 1991). Disease stage and severity could explain these differences in the literature, but as only PD diagnosis GWAS was available to us we cannot speculate on duration or severity.

Our finding that genetically liability to AD and PD does not increase brain iron suggests brain iron is a contributing factor and is not secondary to the disease process. This is also consistent with reports that iron accumulation occurs concomitantly with amyloid accumulation in the brain, as two synergic processes that lead to neurodegeneration (Peters et al., 2015). The possibility that brain iron might be secondary to non-AD dementia disease cannot be excluded because we did not have access to a large independent non-AD GWAS for MR analysis.

This study has several strengths. Our GWAS results confirmed previously reported pathways for brain iron (R2* and QSM): iron transport and homeostasis, metal ion transporters, calcium homeostasis, and ‘glia and myelin’. (Wang et al., 2022) We extend previous reports by analysing the newly discovered variants revealing that other possible pathways such as BMI and blood pressure might affect MRI-estimated iron. This is supported by our observational analysis showing that BMI is a significant predictor of the observed phenotypes. This could bias a MR analysis (due to pleiotropy); however, we demonstrated that genetically predicted brain iron is not associated with BMI or risk of CAD, supporting that the effect of brain iron on non-AD dementia is not via these potential confounders. Additionally, our sensitivity analysis did not suggest any bias due to pleiotropy, following in-depth investigations of the individual variant effects for the thalamus R2* association with non-AD dementia (MR-Egger intercept *p* > 0.05). Therefore our analyses do not appear to violate the 3 core assumptions of MR.

Our study has limitations. Firstly, this volunteer cohort of participants is not population representative (UK Biobank participants are on average healthier; less likely to be obese, smoke, etc) (Fry et al., 2017), meaning dementia may be underestimated. Secondly, dementia diagnoses are ascertained predominantly from hospital inpatient records, meaning diagnoses are likely underestimated. Thirdly, using MR we could only test associations UKB participants genetically similar to the 1000Genomes EUR reference group, as the number of non-EUR participants with MRI data was too small for GWAS. Fourth, MRI-derived iron measures R2* and QSM are estimates of tissue iron: other biological signals such as myelin can influence the estimation (Lee et al., 2021; Ravanfar et al., 2021). Results in myelin-rich brain regions, such as the hippocampus and the thalamus, should be interpreted with caution. Though R2* and QSM are not direct measures of tissue iron, studies have reported their association with sub-cortical brain iron in phantom and postmortem studies (Gustavo Cuna et al., 2023; Langkammer et al., 2012).

In conclusion, our results support a causal effect of higher brain iron in specific sub cortical regions on risk of non-Alzheimer’s dementia and Parkinson’s disease, and grey matter atrophy.

## Supplementary Material

Supplementary Materials

## Figures and Tables

**Table 1:** Summary statistics for the UK Biobank cohort under analysis.

Whole UK Biobank cohort (EUR-like #)		All	Male	Female
Number of participants	N (%)	451,231	206,369 (45.7)	244,862 (54.3)
Age at baseline assessment^~^	Mean (SD)	56.8 (8.0)	57.0 (8.1)	56.6 (7.9)
Follow-up, years *	Mean (SD)	13.3 (2.0)	13.2 (13.2)	13.4 (13.4)
Incident diagnoses:				
All-cause dementia	n (% of N)	8040 (1.78)	4284 (2.08)	3756 (1.53)
Non-AD dementia^	n (% of N)	4246 (0.94)	2480 (1.20)	1766 (0.72)
MRI assessment UK				
Biobank (EUR-like #)				
Number of participants	N MRI (%)	39,533	18,629 (47.1)	20,904 (52.9)
Age at MRI assessment^~^	Mean (SD)	64.3 (7.7)	65.0 (7.8)	63.7 (7.6)
Follow-up, years *	Mean (SD)	4.5 (1.8)	4.5 (4.5)	4.5 (4.5)
Incident diagnoses:				
All-cause dementia	n (% of N MRI)	118 (0.30)	65 (0.35)	53 (0.25)
Non-AD dementia^	n (% of N MRI)	75 (0.19)	44 (0.24)	31 (0.15)
Published GWAS				
Parkinson’s disease^^	N Cases	56,306	NA	NA
Alzheimer’s disease**	N Cases	94,437	NA	NA

# UK Biobank participants with genetic information, genetically similar to the 1000 Genomes EUR reference population.

~ Minimum and maximum age at baseline: 40 to 70 years. Minimum and maximum age at MRI assessment: 45 to 83 years.

* Years between baseline assessment and hospital episode statistics (HES) censoring date (for the majority of participants: 31 Oct 2022) or death, if participant died before this. Minimum and maximum from baseline (for alive participants): 12.1 to 16.6 years. Minimum and maximum from MRI assessment, for alive participants: 0.5 to 5.8 years. Diagnoses are from HES or primary care records, where available (see Methods).

^ Received a dementia diagnosis, excluding participants with an Alzheimer’s- related diagnosis.

** From Kunkle et al. (Kunkle et al., 2019)

^^ From Nalls et al. (Nalls et al., 2019), cases include proxy cases from UK Biobank as described by authors.

## Data Availability

GWAS results are publicly available, but participant level UK Biobank data is only available via application to UK Biobank directly. Full summary statistics are available to download from FigShare (https://doi.org/10.6084/m9.figshare.25343170).
