# Supplementary material for: MRI-derived brain iron, grey matter volume, and risk of dementia and Parkinson’s disease: Observational and genetic analysis in the UK Biobank cohort
Source: Neurobiol Dis. Author manuscript; Available in PMC 2025 Jul 1. (PMC12048010; doi:10.1016/j.nbd.2024.106539)
Supplement: Supplementary Materials [file NIHMS2077411-supplement-Supplementary_Materials.zip › 1-s2.0-S0969996124001384-mmc1.docx]

**MRI-derived brain iron, grey matter volume, and risk of dementia and Parkinson’s disease: observational and genetic analysis in the UK Biobank cohort**

Casanova *et al.*

**Supplementary Information**

[Supplementary Methods 2](#_Toc160544718)

[Dementia ascertainment 2](#_Toc160544719)

[Genotype data 2](#_Toc160544720)

[Mendelian Randomization 2](#_Toc160544721)

[Supplementary Results 4](#_Toc160544722)

[GWAS of 16 IDPs 4](#_Toc160544723)

[Supplementary Figures 5](#_Toc160544724)

[SFigure 1: Putamen QSM to Putamen grey matter volume in all participants 5](#_Toc160544725)

[SFigure 2: Putamen T2* to Putamen grey matter volume in all participants 6](#_Toc160544726)

[SFigure 3: Thalamus T2* to Not-AD dementia in all participants 7](#_Toc160544727)

[SFigure 4: Putamen T2* to not-AD dementia in males only 8](#_Toc160544728)

[References 9](#_Toc160544729)

# Supplementary Methods

## Dementia ascertainment

Dementia diagnoses were ascertained from Hospital Episode Statistics (HES) data from England, Scotland and Wales; censored to 31 October 2022, 31 August 2022, and 31 May 2022, respectively. Diagnoses were also ascertained from the participants death records (to 30 Nov 2022), where the participant had died. Diagnoses in HES and in the death records are coded using International Classification of Diseases version 10 [ICD-10] codes. Primary care (General Practice, GP) data were available in 45% of the cohort (recorded as Read2 or CTV3 codes), up to 2016 or 2017 depending on data provider (https://biobank.ndph.ox.ac.uk/ukb/label.cgi?id=3000).

ICD-10 codes for dementia were taken from the UK Biobank “Definitions of Dementia and the Major Diagnostic Pathologies, UK Biobank Phase 1 Outcomes Adjudication” document (Date: March 2018, Version: 1.0, <https://biobank.ndph.ox.ac.uk/showcase/showcase/docs/alg_outcome_dementia.pdf>). Diagnostic codes for dementia in primary care (Read2 and CTV3) were identified from the UK NHS National Institute for Health and Care Excellence (NICE) Quality and Outcomes Framework (QOF) Business Rules (https://qof.digital.nhs.uk), version 37.0 (version date 09/06/2017).

The specific diagnostic codes used are in Supplementary Table 1.

## Genotype data

Participants were genotyped using two near identical (>95% shared variants, n=805,426 total) microarray platforms: the Affymetrix Axiom UK Biobank array (in 438,427 participants) and the Affymetrix UKBiLEVE array (in 49,950 participants). UK Biobank centrally performed genotype imputation in 487,442 participants using data from the Haplotype Reference Consortium and UK10K reference panels, increasing the number of genetic variants to ~96 million ^1^. We exclude genetic variants with <0.1% minor allele frequency or with imputed INFO score <0.3, leaving 16million for GWAS analysis.

## Mendelian Randomization

The IVW method estimates the causal effect of the genetically predicted exposures on the genetically predicated outcomes ^2^. The IVW assumes there is balanced horizontal pleiotropy (i.e. with a zero mean); we therefore additionally applied ‘weighted median’ (assumes less than 50% of the weight in the analysis comes from invalid instruments) and ‘MR-Egger’ (allows for unbalanced pleiotropy providing genetic variants’ effect on the exposure is not correlated with their pleiotropic effects on the outcome) approaches to test robustness of the estimate. The MR-Egger method additionally estimates an intercept and deviation from the null is used to test for possible unbalanced pleiotropy. We also calculated the $I_{GX}^{2}$ statistic to check for bias due to the `NO Measurement Error’ (NOME) assumption (i.e., where the SNP-exposure association is assumed to be known, rather than estimated). Finally, ‘leave one out’ analysis tests whether the result is overly influenced by a single variant. F-statistics were estimated from betas and standard errors ($\frac{\beta^{2}}{{SE}^{2}}$), as previously reported ^3^.

# Supplementary Results

## GWAS of 16 IDPs

Full summary statistics are available to download from FigShare (https://doi.org/10.6084/m9.figshare.25343170). Across all 16 GWAS we identified 37,835 variants associated with at least one IDP (p<5*10-8), mapping to 250 genomic loci (based on distance pruning using +/-500kb). To determine loci and lead variants we used R package gwasRtools v0.1.4 (<https://github.com/lukepilling/gwasRtools>). The lead variant for each loci, for each IDP GWAS is listed in Supplementary Table 2.

We found substantial inflation of the test statistics ($\lambda_{GC}>1$) which could indicate bias due to cryptic relatedness or population stratification, however in LDSC analysis we found the regression intercepts were all close to 1, indicating this bias is limited.^4^

# Supplementary Figures

## SFigure 1: Putamen QSM to Putamen grey matter volume in all participants


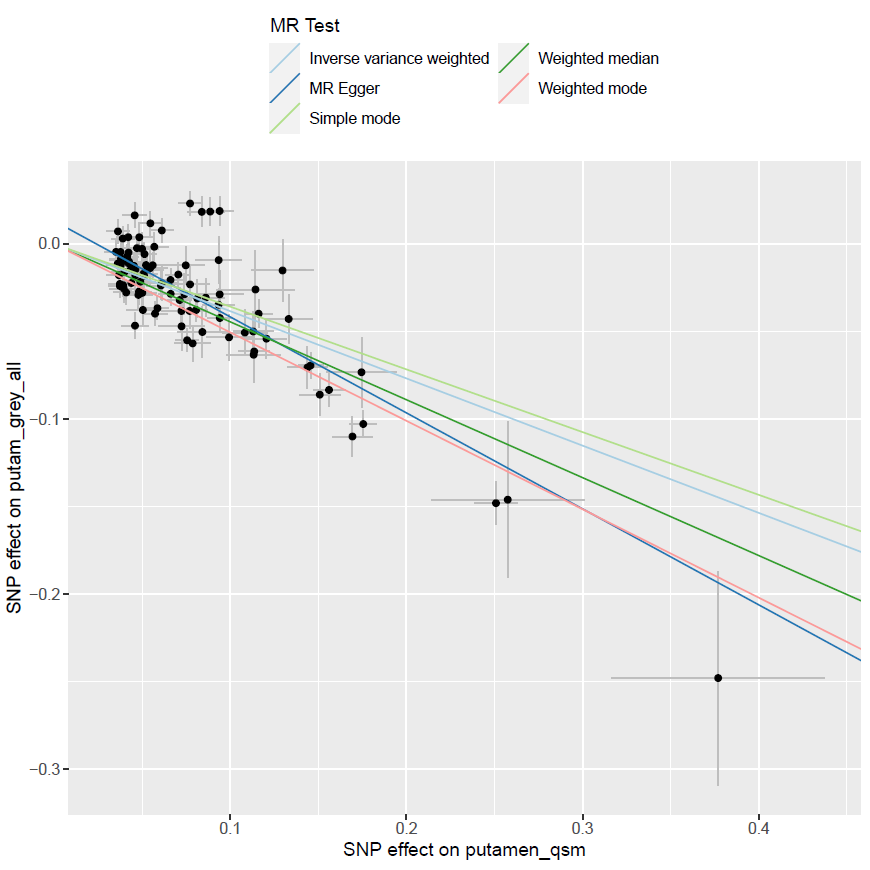


Scatter plot of the SNP-exposure (x-axis) and SNP-outcomes (y-axis) associations from the GWAS results.

## SFigure 2: Putamen T2* to Putamen grey matter volume in all participants


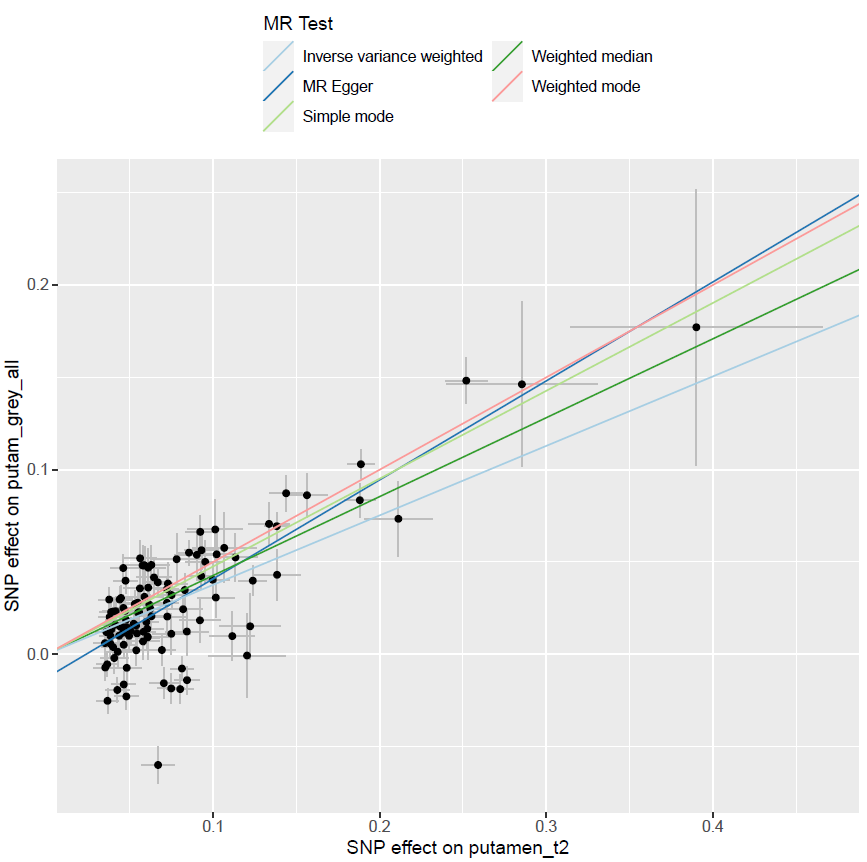


Scatter plot of the SNP-exposure (x-axis) and SNP-outcomes (y-axis) associations from the GWAS results.

## SFigure 3: Thalamus T2* to Not-AD dementia in all participants


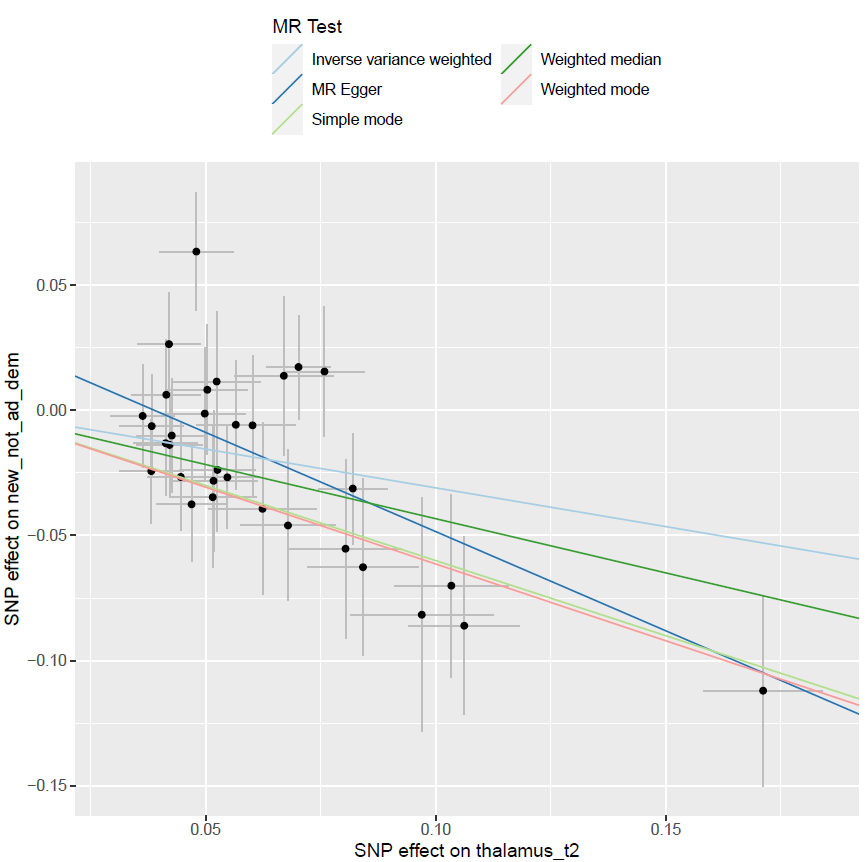


Scatter plot of the SNP-exposure (x-axis) and SNP-outcomes (y-axis) associations from the GWAS results.

## SFigure 4: Putamen T2* to not-AD dementia in males only


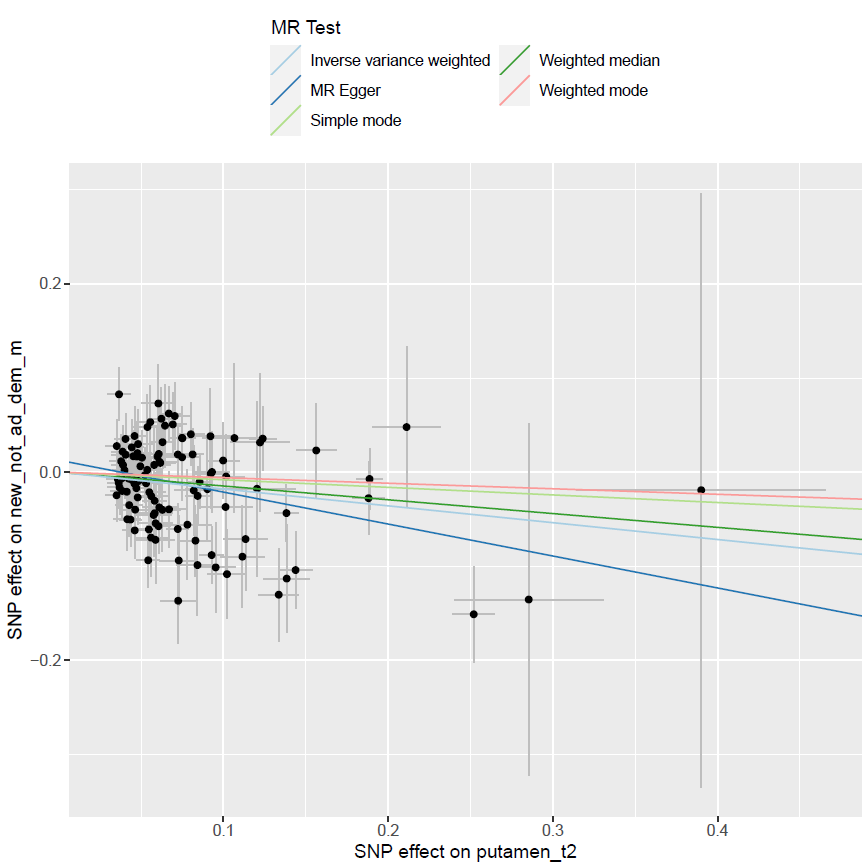


Scatter plot of the SNP-exposure (x-axis) and SNP-outcomes (y-axis) associations from the GWAS results.

# References

1. Bycroft C, Freeman C, Petkova D, et al. The UK Biobank resource with deep phenotyping and genomic data. *Nature* 2018; **562**(7726): 203-9.

2. Burgess S, Scott RA, Timpson NJ, Davey Smith G, Thompson SG. Using published data in Mendelian randomization: a blueprint for efficient identification of causal risk factors. *Eur J Epidemiol* 2015; **30**(7): 543-52.

3. Garfield V, Salzmann A, Burgess S, Chaturvedi N. A Guide for Selection of Genetic Instruments in Mendelian Randomization Studies of Type 2 Diabetes and HbA1c: Toward an Integrated Approach. *Diabetes* 2023; **72**(2): 175-83.

4. Bulik-Sullivan BK, Loh PR, Finucane HK, et al. LD Score regression distinguishes confounding from polygenicity in genome-wide association studies. *Nat Genet* 2015; **47**(3): 291-5.
